# A Systematic Review of Father–Child Play Interactions and the Impacts on Child Development

**DOI:** 10.3390/children8050389

**Published:** 2021-05-13

**Authors:** Erin Louise Robinson, Jennifer StGeorge, Emily Elsa Freeman

**Affiliations:** 1 School of Psychology, University of Newcastle, Callaghan, NSW 2308, Australia; Emily.freeman@newcastle.edu.au; 2 School of Health Sciences, University of Newcastle, Callaghan, NSW 2308, Australia; Jennifer.stgeorge@newcastle.edu.au

**Keywords:** parenting, child development, systematic review, cognition, behaviour, emotion, social, father, child, dyads

## Abstract

Father–child play engagement has been linked to a variety of child developmental outcomes. However, the most prevalent types of play and child developmental outcomes utilised in research remains unclear. The aim of this study was to systematically review the literature on father–child play interactions and the association with child developmental outcomes for children aged 0–10 years. Database searches generated 1622 abstracts that matched the specified search criteria. Abstract screening and full-text review resulted in 39 included publications. The systematic review revealed that while some paternal play behaviours resulted in different impacts across play types, others reported similar impacts. The findings of this review have implications for potential interventions and parenting resources.

## 1. Introduction

From birth, children engage in playful social interactions with their caregivers [1]. Play interactions are typically reciprocal in nature and are based around the idea that parents and their children can work together to seek shared goals [2]. These interactions allow parents to positively foster their children’s cultural learning [3] and provide an avenue for young children to gain a variety of cognitive, emotional, social and behavioural skills [4].

Compared to mother–child interactions, play is more characteristic of the father–child relationship in Western cultures [4]. It has been suggested that fathers spend a greater portion of their time playing with their children than doing any other activity [5]. While both mothers and fathers engage in play with their children [6,7], past research has primarily focussed on maternal influences on child development [8], with only one-third of parent–child play interaction research being conducted with fathers [4]. However, in recent decades, the social movement of involved fatherhood has stimulated a research focus on fathers [9,10]. This has led to an increase in the body of evidence examining the paternal contributions, particular through play, to child development [11,12,13].

Fathers play more often while engaging in caregiving tasks, than do mothers, and their play tends to be more physical, spontaneous and playful [4,14]. Through these challenging play interactions, fathers are able to provide new experiences to their child that mothers might avoid as dangerous (due to differences in parental perceptions of rough-play), while serving as a familiar and safe companion [15,16]. Due to these differences in how parents engage with their children through play, it is unsurprising that research has documented that father–child play makes unique contributions to development, compared with mother–child play interactions [10,17]. These contributions differ particularly in the areas of academic achievement, behavioural and emotional regulation, and cognitive development [18,19].

Although the effects of father–child play interactions seem to be additive, in that both parents make independent contributions to their child’s development [4], there is still much to learn about the specific features of the father–child relationship during play that most strongly impact upon child development. Furthermore, as society pushes for fathers to be more involved in their child’s life [20], a broader knowledge base about the psychological resources a father can provide their children will allow for the facilitation and optimisation of father–child involvement [21].

There are few systematic reviews that have focussed on father–child play. A recent study explored the frequency of play [22], while other research focussed on involvement [23] or chose to focus on a specific play type (i.e., rough-and-tumble play) [24]. However, there has yet to be a systematic review that adopts a broad perspective on the impacts of different kinds of play on child developmental outcomes. By broadening this approach, we stand to gain a more complete picture of the role that father–child play has in child development.

The aim of the present research was to investigate the relationship between father–child play interactions and child developmental outcomes via a systematic review. In addition, we had three research questions of interest:

1. Firstly, we aimed to gain a broad view on the types of play fathers and their children engage in, and by doing so, increase knowledge on the most utilised forms of play throughout paternal research.
2. Secondly, we sought to obtain an understanding of how play is being measured in terms of objective and self-report forms of measurement. Prior research has demonstrated that self-report measures, relative to objective measures, are limited by the responder’s introspective ability, honesty and most notably by response biases [25]. Thus, the purpose of obtaining this information was to determine whether the findings of the reviewed articles should be interpreted with caution
3. Finally, we wanted to better understand which childhood outcomes have been the focal point across these studies.

Based on previous research [10,17], we predicted that when fathers engage in positive parenting behaviours, where they are proactively meeting their children’s needs during play, there would be positive relationships with child developmental outcomes. Conversely, when fathers engage in negative parenting behaviours, consisting of more parent-centred approaches to play where behaviour is not modulated to meet children’s needs [26], it was predicted that this would show negative relationships with child developmental outcomes. The objectives of this research were preregistered with the International Prospective Register of Systematic Reviews (PROSPERO). Further protocol information can be found below.

## 2. Method

### 2.1. Protocol Registration

The protocol outlining the aims and scope of this systematic review was registered with PROSPERO on the 20 November 2018 [27]. The protocol is available from: http://www.crd.york.ac.uk/PROSPERO/display_record.php?ID=CRD42018115301.

### 2.2. Search Strategy

The PsycINFO, Scopus and Web of Science electronic peer-reviewed databases were searched. The search strategy used included key terms relating to father–child play (“father”, “child” and “play) and development (“development”). The key terms were developed within the PsycINFO database and adapted to be used within the other two databases. The search was limited to human studies, with no additional limits used.

### 2.3. Study Selection and Data Extraction

The titles and abstracts obtained from the database searches were screened by two independent reviewers to identify studies that included the following elements: (1) a father and child, (2) a child aged between 0 and 10 years of age, (3) a form of play, and (4) a child developmental outcome. Where there was disparity between the reviewer’s assessments during the review process, a third reviewer was employed for resolution. Inter-rater reliability for this stage was 89%, indicative of a strong level of agreement between reviewers.

After abstract reviews, eligible studies were retrieved for full-text reviews. Two independent reviewers assessed the eligibility of each full-text article for inclusion in the final full-text review. An additional eligibility criterion was included at this stage (5) typically developing children. However, studies that contained non-typically developing children, but contained a control group, were also included. The reason for this inclusion criteria (5) being added for the full-text stage, and not for the abstract stage, was to allow for full-text screening of non-typical developmental studies, where a control group may not have been mentioned in the abstract alone. Akin to the title and abstract review stages, where there was disparity between the reviewer’s assessments during the review process a third reviewer was employed for resolution. Inter-rater reliability for this stage was 94% indicative of a strong level of agreement between reviewers. Reviewers’ reasons for study exclusion were documented during the review process.

Data extraction included details of the sample, methodology and measurement objectivity (child outcome measure and measurement of play) and results (e.g., descriptive and inferential statistics). Play types were categorised based upon the interactions described within each publication. In circumstances where data were not reported in an included study, the author was contacted. Of the three authors contacted, none were able to provide additional information. Data extraction was completed by two reviewers to allow for concurrent resolutions of disagreements.

### 2.4. Assessment of Study Quality

Quality assessment for each included publication was completed by two reviewers. The criteria used included (1) use of a valid and objective measure of the play interaction, (2) use of a valid and objective measure of child development, and (3) sample size acceptable for the statistical analyses utilised. For this research, objective measures were defined as those delivered by the researchers. Thus, self-report measures were not classified as objective. Valid measures were defined as those that had been scientifically validated. Thus, measures that were designed specifically for their respective paper were not classified as valid measures. A score of 0, 1, or 2 was utilised for each criterion (a score of 0 indicated that the criteria were “not satisfied”, a score of 1 indicated that the criteria were “partially satisfied” and a score of 2 indicated that the criteria were “fully satisfied” (see Table 1 for details). An aggregate score was given out of a maximum of 8 points. Quality scores were allocated into categories based upon the following standards: poor = 0–2; fFair = 3–5; good = 6–8.

## 3. Results

### 3.1. Literature Search Process

**Figure 1:**
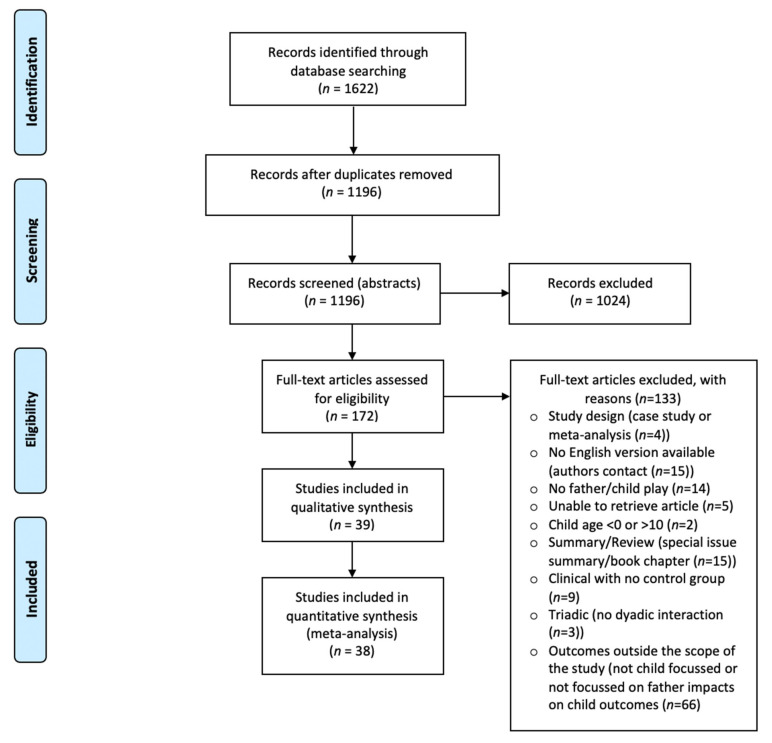
PRISMA flowchart outlining the selection process for the systematic review.

A PRISMA flowchart illustrating the selection process for the systematic review is presented in Figure 1. The initial search contained 1622 abstracts (1196 were unique). During abstract screening, 1024 publications were excluded due to the following reasons: being a case study, a review, a chapter summary, a conference abstract, an animal study, no father child play, no child related outcomes or written in a non-English language. This resulted in 172 publications being retrieved for full-text review. During full-text review, 133 publications were excluded due to the following reasons: inappropriate study design (i.e., case study or meta-analysis), no English version available (where authors were contacted prior to exclusion), no father–child play, article unable to be retrieved (where the article was published some years ago and authors could not provide a full-text version), child beyond the age range of the study (i.e., <0 or >10 years of age), the article was a summary/review (chapter or special issue with no numerical data), clinical population with no control group, triadic mother/father/child interaction with no dyadic interaction between father and child, and outcomes outside scope of the present study (i.e., outcomes were not child focussed or child outcomes not analysed in terms of father involvement or interactions). This resulted in 39 publications containing 39 samples and 246 outcomes being included in this systematic review. All included publications received a total quality score between 6 and 8, indicative of good study quality (see reference list for respective study quality criterion scores).

The play types were examined alphabetically. For this systematic review, child outcome measures were classified as being either positive or negative. Positive child developmental outcomes of interest such as prosocial behaviour, academic achievement and school readiness, emotional regulation and cognitive development were classified as positive outcome measures. Negative child developmental outcomes of interest such as anxiety/withdrawal, anger/aggression, behaviour problems, peer problems and avoidance behaviours were classified as negative outcome measures. Thus, the associations will be presented as a function of the type of outcomes, whereby negative outcome measures and negative associations indicate positive impacts on child development. The results of the included publications are presented in Table 2, Table 3, Table 4, Table 5, Table 6, Table 7, Table 8, Table 9 and Table 10.

### 3.2. System Review: Characteristics and Summary of Results by Play Type

**Figure 2:**
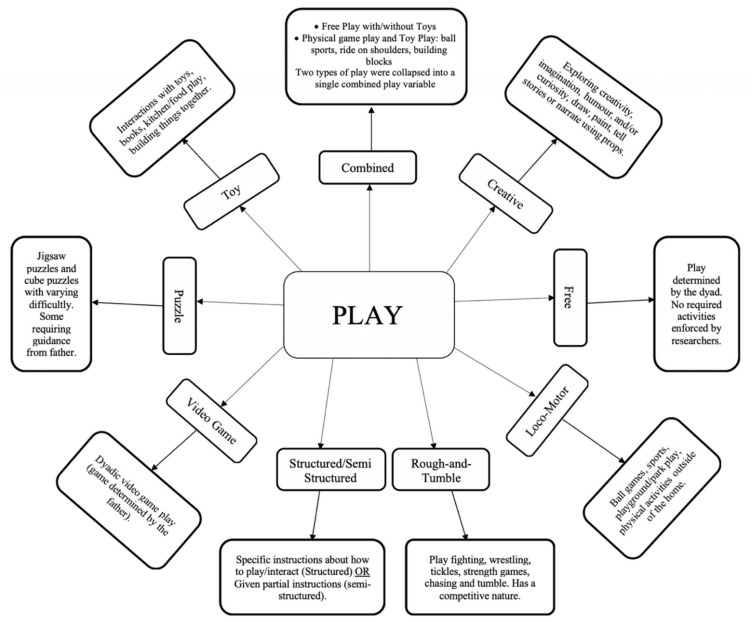
Flowchart providing a brief overview of the various activities found within each play type.

The systematic review resulted in the identification of nine play types: Creative Play, Combined Play (which consisted of the combination of two play types), Free Play, Locomotor Play, Puzzle Play, Rough-and-Tumble Play, Structured and Semi-Structured Play, Toy Play and Video Game Play. The 246 outcomes were separated into their respective play types, where characteristics and results summaries were examined (See Table 2, Table 3, Table 4, Table 5, Table 6, Table 7, Table 8, Table 9 and Table 10). An overview of the activities found within each play type is provided in Figure 2.

Creative Play was examined by three studies, making up 3% of the systematic review outcomes. Child ages within these studies ranged from 2 to 7 years. Half of the Creative Play studies used objective play and child outcome measures, with the other half drawn from parent self-report information (Table 2). The Creative Play studies focussed on the following childhood outcomes: Achievement, in terms of children’s receptive vocabulary (*N* = 1); Emotional/Behavioural, with outcomes inclusive of emotional regulation, withdrawn behaviour, behaviour problems and aggressive behaviours (*N* = 5); and Social/Behavioural, encompassing prosocial behaviours (*N* = 2). Of the eight outcomes, four were interested in negative child outcome measures including child withdrawn behaviour and behaviour problems. Positive associations were found between Creative Play and all Achievement, Emotional/Behavioural and Social/Behavioural outcomes.

Creative Play findings indicated that when fathers undertook positive behaviours during play such as actively engaging their child during the play or being playful, their children showed fewer behaviour problems [28], less aggression [29], better emotional regulation (Emotional/Behavioural) and higher receptive vocabulary (Achievement) [7]. Furthermore, when fathers undertook Creative Play generally, this was positively related to children’s displays of prosocial behaviour (Social/Behavioural) [28].

**Table 2:** Creative play—outcome measure descriptions and results summary.

Study	Sample Size	Outcome Measure	Outcome Category	Number of Reported Positive Associations	Number of Reported Negative Associations
[7]	73	Positive	A	1	0
		Positive	EB	1	0
[29]	87	Negative	EB	0	2
[28]	13,717	Positive	SB	2	0
		Negative	EB	0	2

Note: A = Achievement. EB = Emotional/Behavioural. SB = Social/Behavioural.

Within Combined Play, three studies examined Physical and Toy Play interactions, while one study used Free Play and Toy Play, making up 9% of the systematic review outcomes (Table 3). Child ages within this study ranged from 10 months to 5 years. Within combined play, researchers were more likely to utilise parental self-report to obtain measures of play and teacher reports for child outcomes, with self-report data accounting for 18 of the 22 play measures and 15 of the 22 child outcome measures. The remainder were objective measurements. Five childhood outcomes were explored: Achievement, in terms of language development (*N* = 1); Cognitive, encompassing children’s intelligence and cognitive development (*N* = 3); Cognitive and Social/Behavioural combined outcome (*N* = 1); Emotional/Behavioural, with a comprehensive examination of emotionality and child internalising/externalising behaviours (*N* = 14); and Social/Behavioural outcomes which explored social competency as rated by teachers (*N* = 3), with 10 of the 22 outcomes interested in positive child outcomes.

The study that examined Combined Play and child Achievement [30] found a positive effect, as did the study interested in Cognitive outcomes [31]. Positive associations were found for Combined Play and child Emotional/Behavioural outcomes for 12 of the 15 outcomes, with 3 finding a non-significant negative effect. The results indicated that fathers’ physically active play, within combined play interactions, predicted children’s emotional regulation (Emotional/Behavioural) for high-emotionality children (more sensitive or more emotionally reactive) but did not predict emotional regulation for low-emotionality children (less emotionally reactive to a stimulus) [32].

When considering Social/Behavioural outcomes and Combined Play, one study reported that father play positively predicted children’s social outcomes [32], while three outcomes suggested that father involvement was negatively associated with child social competency [33]. These negative findings indicated that the more that the father was involved in play, the less social competency the child showed.

**Table 3:** Combined play—outcome measure descriptions and results summary.

Study	Sample Size	Combined Play Types	Outcome Measure	Outcome Category	Number of Reported Positive Associations	Number of Reported Negative Associations
[32]	727	P-T	Positive	SB	1	0
			Positive	EB	1	1
[31]	14	P-T	Positive	C	2	0
[33]	112	P-T	Positive	SB	0	3
			Negative	EB	2	10
[30]	97	F-T	Positive	A	1	0
			Positive	C	1	0

Note: A = Achievement. C = Cognitive. EB = Emotional/Behavioural. SB = Social/Behavioural. F−T = Free and Toy Play. P−T = Physical and Toy Play.

Free Play accounted for the smallest portion of outcomes of the systematic review data, with only three studies examining this type of play, making up 2% of the systematic review outcomes (Table 4). Child ages within this study ranged from 1 to 3 years. All of the studies used objective measurements for both play and child outcomes measurements and were interested in positive child outcomes. Free Play researchers were interested in child Achievement and Emotional/Behavioural outcomes, with all studies finding positive associations. Achievement outcomes encompassed receptive and general language, while the Emotional/Behavioural outcome involved child emotional regulation.

**Table 4:** Free play—outcome measure descriptions and results summary.

Study	Sample Size	Outcome Measure	Outcome Category	Number of Reported Positive Associations	Number of Reported Negative Associations
[34]	175	Positive	A	1	0
[35]	34	Positive	A	2	0
[36]	90	Positive	EB	1	0

Note: A = Achievement. EB = Emotional/Behavioural.

Free Play findings demonstrated that father positive parenting behaviour during play was positively associated with child outcomes; nurturance was positively associated with child receptive language (Achievement) [34], and sensitive regulation was positively associated with child-regulation compliance (Emotional/Behavioural) [36]. Further positive associations were found for father didactics (father teaching his child) and his child’s language development (concurrently and predictive of) [35].

Six studies examined Locomotor Play in their research, making up 11% of the systematic review outcomes (Table 5). Child ages within this study ranged from 9 months to 7 years. Researchers gathered parent self-report information to obtain Locomotor Play measurements for 17 of the 26 studies, with the remainder obtained from objective measurements. However, for child outcome measurements, 73% of outcomes came from objective measures, while the remainder were obtained from parental self-report. A large portion of outcome measures were focussed on Emotional/Behavioural child outcomes (*N* = 11), with the other areas of interest spread between Achievement (*N* = 8), Cognitive (*N* = 4), and Social/Behavioural outcomes (*N* = 3). The Emotional/Behavioural outcomes explored behavioural problems, child anxiety/withdrawal, anger-aggression, internalising behaviours, child temperament, self-regulation, behaviour problems and socio-emotional functioning. Achievement outcomes included literacy, mathematics and school readiness. Cognitive child outcomes incorporated executive functioning and cognitive development and Social/Behavioural outcomes explored prosocial behavioural and social competence. The vast majority of outcomes were concerned with positive child outcome measures (*N* = 22).

Within the study interested in child achievement, negative associations were found for four of the eight outcomes, with father overstimulation during play resulting in negative childhood achievement outcomes [37]. For Locomotor Play and child Cognitive outcomes, there were an equal number of positive (*N* = 2) and negative associations (*N* = 2) reported, with one study suggesting that father overstimulation during Locomotor Play resulted in poorer scores of executive functioning [37], while another reported mixed findings between paternal Locomotor Play and child cognitive development [38].

Positive associations were found in all studies that measured child Social/Behavioural outcomes [28,39]. Of the 12 Emotional/Behavioural child outcomes, associations were mixed—1 found no effect, while 5 reported negative associations and 6 reported positive associations. Quality of play was positively associated with lower internalising scores [6], while father’s involvement in play was positively associated with lower risks of behaviour problems [28] and aggression (Emotional/Behavioural) [39]. Negative associations were reported between paternal Locomotor Play and socio-emotional functioning, child temperament and self-regulation [38].

**Table 5:** Locomotor play—outcome measure descriptions and results summary.

Study	Sample Size	Outcome Measure	Outcome Category	Number of Reported Positive Associations	Number of Reported Negative Associations
[6]	103	Negative	EB	0	1
[40]	750	Positive	A	2	0
[28]	13,717	Positive	SB	2	0
		Negative	EB	0	2
[38]	3770	Positive	C	1	1
			EB	0	6
[37]	89	Positive	A	2	4
			C	1	1
[39]	295	Positive	SB	1	0
		Negative *	EB	0	3

Note: A = Achievement. C = Cognitive. EB = Emotional/Behavioural. SB = Social/Behavioural. * = One outcome showed no effect.

Puzzle play was examined by two studies, making up 9% of the systematic review outcomes (Table 6). Child ages within this study ranged from 3 to 5 years. Objective measures were obtained for all play and child outcomes. These studies focussed on child Achievement (*N* = 13) and Cognitive outcomes (*N* = 10). All outcomes were positive child outcome measures (*N* = 23). The Achievement outcomes were interested in literacy, school readiness and mathematics and the Cognitive outcome of interest was child executive functioning.

For child Achievement, positive associations were found for 8 of the 13 outcomes, with father control (negative parenting behaviour) during puzzle play resulting in negative childhood achievement outcomes [37,41]. Results showed a positive association between fathers who supported child autonomy during play (positive parenting behaviour) and child vocabulary [37,41], mathematic achievement and school readiness [37]. For child Cognition, positive associations were found for 6 of the 10 outcomes, with father control during puzzle play resulting in negative childhood executive functioning outcomes [41]. Father autonomy support (positive parenting behaviour) was associated with positive executive functioning outcomes [37]. Results demonstrate that the way in which fathers choose to engage positively by fostering their children’s autonomy or negatively by inhibiting their autonomy (control), results in different developmental outcomes for children.

**Table 6:** Puzzle play—outcome measure descriptions and results summary.

Study	Sample Size	Outcome Measure	Outcome Category	Number of Reported Positive Associations	Number of Reported Negative Associations
[41]	110	Positive	A	1	1
			C	2	4
[37]	89	Positive	A	7	4
			C	4	0

Note: A = Achievement. C = Cognitive.

Rough-and-Tumble Play (RTP) was examined in nine studies, making up 24% of the systematic review outcomes (Table 7). Child ages within this study ranged from 9 months to 8 years. Objective measures were gathered for 46 of the 58 rough-and-tumble play measures, with the remainder obtained from parent self-report measurements. However, for child outcome measurements, the reverse was seen with 82.76% of child outcomes acquired through teacher, peer and parent self-report, with only 17.4% of child outcomes measured objectively. Within the RTP literature, the largest portion of outcomes were focussed on child Social/Behavioural functioning (*N* = 31), followed by Emotional/Behavioural outcomes (*N* = 26), with only one reported child Cognition outcome (1). There was a similar spread of positive (*N* = 32) and negative child outcome measures (*N* = 26). For Cognitive outcomes, there was a positive effect between child cognitive scores and father–child RTP [42]. The Emotional/Behavioural outcomes of interest fell broadly across child physical aggression, verbal aggression, conduct problems, total emotional/behavioural problems, emotional problems, hyperactivity problems, anger/aggression, emotional regulation and anxiety/withdrawal. Of these, the studies reported 16 positive associations between RTP and child outcomes and 8 negative associations. Within the negative associations RTP frequency was positively correlated with child physical aggression when fathers were less directive in play [43,44], and negatively correlated with emotional regulation when fathers were less dominant in play [45]. Furthermore, negative associations were found for challenging parenting behaviours and child anxiety [46], and reciprocal negative affect during play was positively associated to children’s verbal aggression [43]. However, other findings reported that involvement in RTP reduced anger/aggression [45,47] and anxiety/withdrawal [39].

The Social/Behavioural child outcomes of interest were social competence, social acceptance, prosocial behaviour, sharing, avoidance and peer problems. A greater number of negative associations were reported between RTP and Social/Behavioural child outcomes, with 19 negative associations compared with 12 positive associations. Of the negative associations, 68% reported that negative affect during RTP (father negative affect or reciprocal negative affect) resulted in various poor Social/Behavioural outcomes such as lower peer rating, social acceptance, and sharing [48]. Interestingly father positive affect during play was associated with negative teacher and peer ratings of social acceptance for girls, and negatively associated with teacher ratings of social acceptance for boys [48]. In addition, father RTP scores and father involvement in RTP were negatively associated with prosocial behaviour and social competence, respectively [48]. A further negative association was found between quality of RTP and child prosocial behaviour [49].

**Table 7:** Rough-and-tumble play—outcome measure descriptions and results summary.

Study	Sample Size	Outcome Measure	Outcome Category	Number of Reported Positive Associations	Number of Reported Negative Associations
[47]	42	Negative	EB	0	2
[42]	1099	Positive	C	1	0
[43]	41	Positive	SB	0	2
		Negative	EB	4	0
[44]	85	Negative	EB	1	2
[45]	34	Positive	EB	1	2
		Negative	EB	2	4
[49]	26	Positive	SB	0	1
		Negative	EB	0	5
[48]	116	Positive	SB	15	9
[46]	132	Negative	EB	1	3
[39]	295	Positive	SB	0	1
		Negative	EB	0	2

Note: C = Cognitive. EB = Emotional/Behavioural. SB = Social/Behavioural.

Structured and Semi-Structured Play was examined by two studies, making up 4% of the systematic review outcomes (Table 8). Child ages within this study ranged from 2 to 10 years. Objective measures were gathered for all play outcomes and 8 of the 13 child outcomes. The remaining five child outcomes were acquired through parent self-report measures. Across the Structured and Semi-Structured Play studies, three outcome categories of interest were identified: Achievement (*N* = 6), Cognitive (*N* = 2) and Emotional/Behavioural outcomes (*N* = 5). The Achievement outcomes were concerned with child literacy and numeracy, with positive associations being found between paternal cognitive stimulation (attempting to further their child’s learning and understanding) during semi-structured play and all Achievement outcomes [50]. For Cognitive outcomes, child cognitive ability was investigated, with paternal cognitive stimulation during semi-structured play showing positive associations for cognition [50]. The Emotional/Behavioural outcomes were child negative affect, conduct problems, emotional symptoms, surgency and effortful control with three negative and two positive associations reported. Of the negative child outcomes, parental sensitivity during play was positively associated with child negative affect and emotional symptoms, and negatively associated with child conduct problems. For the positive child outcomes, a positive effect was found between father sensitivity during play child effortful control, while father sensitivity during play was negatively associated with child surgency [51]. This conveys that while sensitivity seemed to positively impact child emotions, it conversely negatively impacted child temperament, which establishes how we react to that emotion. Furthermore, as surgency is a personality trait which conveys cheerfulness, spontaneity and extraversion, and effortful control dictates how well a child has self-regulation over their emotional reactivity and behaviour, sensitivity appears to improve children’s skills in controlling their reactions, which results in lowering impulsiveness and outgoingness.

**Table 8:** Structured and semi-structured play—outcome measure descriptions and results summary.

Study	Sample Size	Outcome Measure	Outcome Category	Number of Reported Positive Associations	Number of Reported Negative Associations
[51]	107	Positive	EB	1	1
		Negative	EB	2	1
[50]	229	Positive	C	2	0
			A	6	0

Note: A = Achievement. C = Cognitive. EB = Emotional/Behavioural.

Twelve studies examined Toy Play in their research, making up 28% of the systematic review outcomes (Table 9). Child ages within this study ranged from 1 to 4 years. Objective measures were obtained for all 68 Toy Play measures and 63 of the children’s outcome measures, while 5 self-report measures of children’s outcomes were utilised. Within the Toy Play literature, the largest portion of outcomes were focussed on Cognitive Outcomes (*N* = 24), closely followed by child Achievement Outcomes (*N* = 23) and Emotional/Behavioural outcomes (*N* = 15), with six reported child Social/Behavioural outcomes (*N* = 6). There were more positive (*N* = 57) than negative child outcome measures of interest (*N* = 11).

The Cognitive outcomes of interest were cognitive development and cognitive flexibility components of executive functioning and mental development. A total of 16 positive associations and 8 negative associations were found. Fathers’ engagement in Toy Play [52], paternal sensitivity [12], fathers responsive-didactic behaviour [53] and paternal positive regard [54] were all associated with positive outcomes, while father detachment, negative regard and negative intrusiveness were associated with negative outcomes in terms of children’s mental developmental index scores [54].

Achievement outcomes of interest were language complexity, expressive communicative compliance, receptive language ability, math achievement, language development, receptive vocabulary, with more positive associations (*N* = 17) found than negative associations (*N* = 6). Fathers’ play behaviour [55,56], mutual compliance [57], high supportiveness [58], dyadic reciprocity [59], sensitivity, cognitive stimulation and positive regard [54] were all associated with positive associations. Negative associations were found between father detachment, intrusiveness and negative regard, and children’s receptive vocabulary [54].

The Emotional/Behavioural outcomes were concerned with child minimum engagement of self-control with forbidden toys and child active engagement of self-control (interacted with forbidden toys less), child aggression, percentage of night sleep, emotional regulation and child negativity. Thirteen of the Emotional/Behavioural associations were positive while two were negative. Shared positive emotion, mutual compliance [59], playfulness [12], quality of interactions [60] and engagement in toy play [56] were factors associated with positive associations. Of the negative associations, dyadic reciprocity during toy play was negatively associated with children’s minimum and active engagement of self-control (showed less self-control with forbidden toys) [59]. Thus, regardless of the positive shared experiences during Toy Play, children still ignored experimenter instructions and engaged in a play with a forbidden toy but were likely to follow their fathers’ verbal instructions. This demonstrates the impact that these dyadic experiences have on the relationship between father and child but may indicate this compliance does not extend to outside parties.

The Social/Behavioural child outcomes of interest were prosocial behaviour, child-friend interactions, friendship quality and false belief understanding. Positive associations were found for all outcomes. Father–child dyads who engaged in more mutual compliance (dyadic measure) and shared more positive emotion during play had children who were more prosocial [57] and father sensitivity showed positive outcomes for child-friend interactions, friendship quality and false belief understanding [61]. Furthermore, mutual compliance and sharing positive emotions during toy Play were negatively associated with child aggression and positively associated with prosocial behaviours (Social/Behavioural) [57].

**Table 9:** Toy play—outcome measure descriptions and results summary.

Study	Sample Size	Outcome Measure	Outcome Category	Number of Reported Positive Associations	Number of Reported Negative Associations
[52]	62	Positive	C	1	0
[60]	70	Positive	EB	1	0
[59]	80	Positive	A	6	0
		Positive	EB	0	2
		Negative	SB	0	4
[57]	88	Positive	SB	2	0
		Negative	SB	0	2
[55]	60	Positive	A	1	0
[12]	111	Negative	SB	0	4
[58]	200	Positive	A	2	0
[61]	32	Positive	SB	3	0
		Negative	SB	1	0
[56]	74	Positive	A	2	0
			C	2	0
			EB	2	0
[53]	65	Positive	C	2	0
[54]	111	Positive	A	8	4
			C	9	8
[62]	620	Positive	C	2	0

Note: A = Achievement. C = Cognitive. EB = Emotional/Behavioural. SB = Social/Behavioural.

One study [63] used Video Game Play to examined childhood outcomes, making up 10% of the outcomes within this systematic review (Table 10). Child ages within this study ranged from 4 to 6 years. Objective measures were gathered for all video game play measures and child outcome measurements. Within this study, outcomes were focussed on Social/Behavioural outcomes (*N* = 16) and Emotional/Behavioural outcomes (*N* = 8). The Social/Behavioural outcomes of interest included eight positive child outcomes and focussed on positive parallel play with peers, while the eight negative child outcomes, focussed on negative peer play (a negative atmosphere with one play partner dissatisfied with the play). For the Social/Behavioural outcomes there were seven positive associations reported. Five positive associations were reported between father factors in Video Game Play inclusive of derisive humour (mocking/ridicule during play), criticism, enthusiasm, affection and father engagement and child outcome of positive parallel play with peers (side-by-side play where both parties are playing separately with neutral affect), while both engagement and derisive humour were negatively associated with negative peer play. Of the nine negative associations reported for Social/Behavioural outcomes, father enthusiasm, affection, intrusiveness, commands, responsiveness and criticism during play were positively related to negative peer play, while intrusiveness, commands and responsiveness also showed negative associations for positive parallel play with peers. The Emotional/Behavioural outcome of interest was positive affect during peer play. Researchers reported two positive associations and six negative associations. The positive associations were found between father affection and father responsiveness and child outcomes of positive affect during peer play. Father engagement, commands, intrusiveness, derisive humour, criticism, and enthusiasm during play were associated with negative associations on positive affect during peer play.

**Table 10:** Video game play—outcome measure descriptions and results summary.

Study	Sample Size	Outcome Measure	Outcome Category	Number of Reported Positive Associations	Number of Reported Negative Associations
[63]	24	Positive	EB	2	6
			SB	5	3
		Negative	SB	6	2

Note: EB = Emotional/Behavioural. SB = Social/Behavioural.

## 4. Discussion

The systematic review revealed that there were nine play types that fathers engaged in with their children: Creative Play, Combined Play, Free Play, Locomotor Play, Puzzle Play, Rough-and-Tumble Play, Structured and Semi-Structured Play, Toy Play and Video Game Play. Upon further investigation, it was apparent that the most utilised forms of play throughout the studies fell across two play types: Toy Play and RTP, with twelve and nine studies, respectively, focussing on these types of play. These two play types accounted for over half of the studies included in the systematic review. This play type bias, may be representative of the types of play that researchers themselves believe to be the most utilised by fathers, perpetuating the idea that the scope of father–child interactions are limited.

This systematic review also uncovered the childhood outcomes that were the focal points of these studies. Emotional/Behavioural outcomes were included in 22 studies, Cognitive and Achievement outcomes were each included in 12 studies and Social/Behavioural outcomes were included in 10 studies. Consequently, it is apparent that past research has primarily focussed on how play impacts children’s emotional and behavioural development. This highlights the need to explore how paternal play impacts cognition, achievement and their social interactions, as these areas have been overlooked.

It was found that the vast majority of included publications focussed on positive child developmental outcomes (75%). While some play types had a relatively even spread of positive vs. negative outcomes of interest (Creative Play, Combined Play and RTP) others focussed largely (Locomotor Play, Structured and Semi-Structured Play, Toy and Video Game Play) or completely (Free Play and Puzzle Play) on positive child developmental outcomes. This may be indicative of a research tendency to illuminate how paternal behaviour is related to positive outcomes for children, rather than determining what paternal behaviours contribute to negative developmental outcomes.

**Figure 3:**
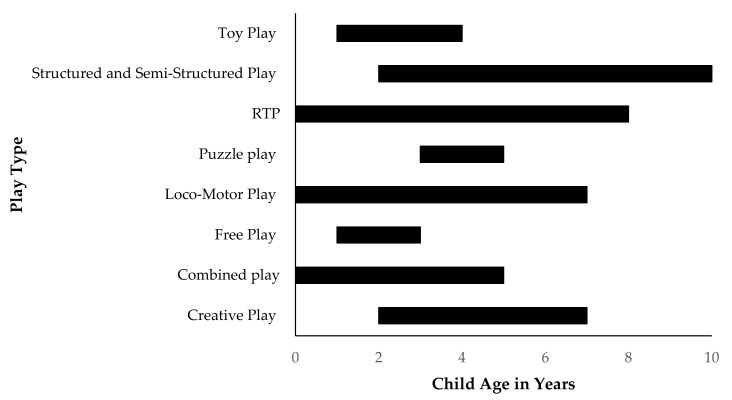
Child age ranges across play types for included publications.

The ages within this systematic review varied across the play types (see Figure 3). What stands out is the general trend towards investigating the younger years of child development. All play types considered the development of children aged 3 years. Four of the play types included samples of children over the age of 5 and only two included children over 7 years. Given these publications investigated father–child play and child development, there may be a neurological rationale for the focus on younger children. Neurological development is critical within the first 5 years of life, where experiences and practice give rise to rapid change and growth (neuroplasticity) [64]. Research targeting a time of rapid development and paternal behaviour may stand to positively inform parenting practices, thus providing opportunities to benefit child development. In line with this, the Structured and Semi-Structured Play study that considered 10-year-old children was examining the longitudinal developmental effects of play at age 2. Additionally, the RTP study that considered 8-year-old children was a five-year follow-up study from play at age 3. Therefore, it appears that when older samples were included, this was to examine the enduring impacts of father–child play, not the concurrent impacts.

It was also found that the measures used for play outcomes were comprised mostly of objective measures (79.27%), while the remainder came from parental self-report. This is reassuring as it indicates that the play outcomes measured within this systematic review have utilised primarily objective measures, which indicates the outcomes are an accurate and unbiased reflection of the various components of these dyadic interactions [65,66]. The child developmental outcomes also utilised a high percentage of objective measures (65.45%), with parent ratings the subsequently most common measure (19.90%), followed by teacher ratings (12.20%) and combined parent-teacher ratings (2.45%). This is indicative of the reports needed for the outcomes themselves, as some outcomes can be obtained objectively in one research session, whilst others unavoidably require parental input to obtain a holistic view of the child’s functioning [67].

Creative Play, Combined Play, Free Play, Structured and Semi-structured Play and Toy Play were all found to be related to child academic achievement outcomes. These different play types, while focussed on unique paternal and dyadic elements of the play, held a common undertone of positive interaction elements. For example, positive relationships were found between Paternal Playfulness (Creative Play), Sensitivity (Combined Play), Nurturance and Dyadic interactions (Free Play), Cognitive stimulation (Structured and Semi-Structured) and dyadic reciprocity, shared positive emotion, mutual compliance, supportiveness, positive regard (Toy Play) and achievement outcomes. This is encouraging as it demonstrates that positive achievement outcomes are not exclusive to a single play type, but instead show that fathers being attuned to their children’s needs, interacting in a playful and stimulating manner, and being supportive of their children’s needs, consistently foster positive relationships. This could be utilised in future parenting interventions. For example, by encouraging positive play interactions within father–child dyads, there is the potential for school academic outcome improvement.

RTP, Locomotor Play and Puzzle Play were all related to positive Cognitive outcomes. General play involvement (RTP and Locomotor Play), Father involvement and autonomy support (Puzzle Play) were play elements of interest that showed positive relationships with child Cognition outcomes. All these types of play share a common factor in terms of gross and fine motor skills. As motor development impacts on child exploration of their physical environment, which in turn effects cognition, this is an instinctual connection [68,69]. These findings are promising as there has been no research linking puzzle play to the physical elements of RTP or Locomotor Play, which are physical in nature. These findings provide prospective benefits for low-income families, where access to puzzles may not be possible, allowing them to derive comparable cognitive developmental outcomes for their children through more vigorous play activities. Across the 58 RTP outcome measures, only 1 outcome looked at cognition. Thus, given these findings and that research into RTP has primarily focussed on behavioural outcomes, it is paramount that further research is invested into exploring the cognitive benefits of RTP.

The systematic review suggested that Video Game Play, along with Creative Play and Toy Play, was related to child social/behavioural outcomes. Father enthusiasm, affection, engagement, responsiveness (Video Game Play), general play involvement (Creative Play), sensitivity and mutual compliance were all positively related to child social/behavioural outcomes in terms of positive interactions with their friends and general prosocial behaviours. This demonstrates the importance of modelling the appropriate ways of engaging in social situations. By being amenable, sensitive and responsive to their child’s needs during play, fathers demonstrate the correct ways for their children to engage with their peers. Furthermore, by fathers showing enthusiasm and engagement in what their play companion (child) is doing, children appear to transfer the same reverence to their peers. Thus, while these three types of play differ in terms of the activities that they involve, it is apparent that strong translational learning can occur during dyadic play, which can foster positive social relationships for children. This modelling has been well described in Bandura’s social learning theory whereby children observe models (people), translate this behaviour and subsequently imitate this learning behaviour [70]. The importance of this is that both positive and negative behaviours can be imitated, thus it is important that fathers are fostering positive social interactions for their children to model.

Creative Play, Free Play, Structured and Semi-Structured Play and Toy Play were all found to be related to child emotional/behavioural outcomes. Sensitivity (Structured and Semi-Structured Play, Free Play), general play (Toy Play) and playfulness (Creative Play) all attained positive outcomes, notably in the area of emotional regulation. This is interestingly contrasted with RTP and Locomotor Play, which showed that general play (Locomotor) and play frequency (RTP) were negatively associated with emotional regulation. This poses the question as to whether there is more nuance in physical play than other play types? Past research has suggested that it is not simply enough to engage in RTP, but instead it needs to be a quality interaction [49]. For example, sharing the winning and losing, sharing of dominance during play and, as there is an element of competition within RTP, fathers praising the child for their efforts. Thus, it is possible that these physical interactions obtained in this review were not quality interactions. Furthermore, as RTP has focussed mainly on behavioural outcomes it is evident that additional exploration is needed to better understand the elements of play that constitute high quality play and thus provide positive impacts to children. By gaining this understanding, we can generate resources for parents, educating them on the positive ways to engage in physical play to ensure beneficial outcomes for their children.

The directions of the relationships between paternal play and child developmental outcomes were in the trend we would expect and in line with our predictions, with the majority of negative outcomes having negative associations reported (77.27%), indicative of positive impacts on child development. For positive outcomes 64.25% found positive impacts on child development. It is important to note that of the negative associations reported, 71% came from negative parenting behaviours such as paternal overstimulation, negative affect, detachment, negative regard, intrusiveness, control, criticism and commands. Thus, consistent with what we would anticipate from these relationships. The positive parenting behaviours that were negatively associated included play involvement, play frequency, engagement, responsiveness, enthusiasm and dyadic reciprocity. As previously stated merely being involved in play does not constitute high quality play [49], thus other unmeasured aspects of the play could be impacting on these associations.

This study has potential limitations. Firstly, due to the broad age ranges considered within this review, the variance in age ranges found for each play type may be problematic. Some play types demonstrated relatively narrow age ranges (Free Play 1–3 years, Puzzle Play 3–5 years, Toy Play 1–4 years, Video Game Play 4–6 years) while others displayed large age ranges (Creative Play 2–7 years, Combined Play 10 months–4 years, Locomotor Play 9 months–7 years, RTP 9 months–8 years, Structured and Semi-Structured Play 2–10 years). As participation in play interactions have been shown to differ across child developmental periods [71] the different age ranges shown here may affect the generalisability of these findings. While this review does not consider findings within a particular developmental lens, future reviews may consider limiting their searches to a more focussed developmental period.

Secondly, the decision to consider all play types within this review subsequently resulted in a small sample of studies within each play type. Consequently, relatively few studies explored the same play/outcome relationships. Despite this, the consideration of all play types allowed for a comprehensive exploration of how father–child play influences child development. It enabled us to answer our research question regarding the types of play fathers and their children engage in, thus providing information about what forms of play are utilised throughout paternal research (Locomotor Play, RTP and Toy Play). A narrower approach for future research may highlight important outcome similarities and/or differences in specific play types. This could allow researchers to form stronger conclusions about the relationship between a chosen play type and a particular developmental outcome.

In addition, there remain opportunities to explore father–child play from a cross-cultural perspective. The majority of this research has been conducted in Western-individualist populations [21,44] and has not explored these interactions in individualist cultures where father–child interactions may differ [72].

Limitations of systematic reviews more broadly are publication biases (less likely to publish no effect findings) and outcome reporting biases (reporting favourable relationships) [73]. However, the articles obtained reported both favourable and unfavourable results. Thus, while potential publication biases herein may have implications in distorting the true picture of the paternal play/child outcome relationship, outcome reporting bias has not surfaced as a concern for the present research.

The results of this systematic review provide support for a relationship between father–child play interactions and child developmental outcomes. It highlighted the broad range of play types utilised throughout father–child play research (Creative Play, Combined Play, Free Play, Locomotor Play, Puzzle Play, Rough-and-Tumble Play, Structured and Semi-Structured Play, Toy Play and Video Game Play) and identified that play outcomes were measured primarily objectively within the reviewed articles. It was made apparent that the principal outcome of interest in these articles was Emotional/Behavioural outcomes, followed by Cognitive and Achievement outcomes and finally Social/Behavioural outcomes.

The results also demonstrated how the same paternal behaviour can have vastly different associations with child outcomes, both within the same play type and across play types. Additionally, the findings highlighted the need to broaden our understanding about seemingly positive and negative parenting behaviours, as the directions of the relationships were at times unexpected, emphasizing the complexity of dyadic interactions and their associated outcomes. They demonstrated that while particular paternal behaviours may have negative impacts for certain childhood outcomes, the same behaviour can have various positive impacts also. Nonetheless, the overall directions of the relationships between paternal play and child developmental outcomes were in the direction we would expect for both positive and negative parenting behaviours. These findings encourage the further exploration of different types of paternal play interactions.

## Figures and Tables

**Table 1:** Point allocations for study quality criterion.

Criterion	0 Points	1 Points	2 Points
(1) Play interaction measure	Neither objective nor validated	Objective or validated	Objective and validated
(2) Child outcome measure	Neither objective nor validated	Objective or validated	Objective and validated
(3) Sample size appropriate for the analysis?			
Means Regression Correlation	<15 per group <10 per predictor <30	15–30 per group 10–20 per predictor 30–50	>30 per group >20 per predictor >50
(4) Sufficient data reported	Insufficient for meta-analysis AND not provided by author on request	Insufficient for meta-analysis but provided by author on request	Sufficient data for meta- analysis included in the publication

Note: Scores ranged from 0 to 8. Categories were applied based on score standards: poor = 0–2, fair = 3–5, and good = 6–8.

## Data Availability

Results are based on public data from the included studies.
